# Outcome and Prognostic Factors in T4a Oropharyngeal Carcinoma, Including the Role of HPV Infection

**DOI:** 10.1155/2014/390825

**Published:** 2014-03-31

**Authors:** Georgios Psychogios, Konstantinos Mantsopoulos, Abbas Agaimy, Kathrin Brunner, Elisabeth Mangold, Johannes Zenk, Heinrich Iro

**Affiliations:** ^1^Department of Otorhinolaryngology, Head and Neck Surgery, Friedrich Alexander University of Erlangen-Nuremberg, Waldstraße 1, 91054 Erlangen, Germany; ^2^Institute of Pathology, Friedrich Alexander University of Erlangen-Nuremberg, Krankenhausstraße 10, 91054 Erlangen, Germany

## Abstract

*Background*. The prognosis of patients with advanced oropharyngeal carcinoma (OPSCC) is generally poor. The aim of this study is to investigate the different therapeutic approaches and identify prognostic factors associated with a worse outcome for patients treated for T4a OPSCC, in order to improve treatment selection for the individual. *Methods*. A retrospective study was conducted on 426 patients with T4a OPC treated between 1980 and 2010. Eleven prognostic factors including treatment modality, lymph node staging, and p16 status as a surrogate marker for human papillomavirus (HPV) infection were analyzed. *Results*. Univariate analysis showed a significant difference in DSS between N0 and N+ (57.1% versus 26.9%, *P* < 0.001), primary surgical and primary nonsurgical treatment (52.7% versus 31.4%, *P* < 0.001), and perinodal invasion (51.7% versus 19.9%, *P* = 0.011). P16-negative patients tended towards a worse DSS than p16-positive patients (40.2% versus 64.6%, *P* = 0.126) but responded better to primary surgery than to nonsurgical treatment (71.4% versus 34.0%, *P* = 0.113). Multivariate analysis identified the N category as an independent prognostic factor for survival. *Conclusion*. The survival of p16-negative patients was worse than p16-positive patients, although they seem to respond better to primary surgery. The strongest independent prognostic factor for T4a carcinomas proved to be the presence of lymph node metastases.

## 1. Introduction

The management of patients with locally advanced oropharyngeal squamous cell carcinoma (OPSCC) has evolved greatly. A decade ago, several studies showed that radiotherapy (RT) in combination with chemotherapy (CT) offers oncologic and functional results similar to those of surgery but with lower severe complication rates [1–3]. Furthermore, in the light of increasing importance of human papillomavirus (HPV) infection in OPSCC and better survival after radiochemotherapy (RCT) in this group of patients, primary RCT has emerged as treatment of choice for this subset of patients in many institutions [4, 5].

However, recent studies showed that both RT and CT can cause serious morbidity such as dysphagia, mandibular osteoradionecrosis, and pharyngeal strictures and may be associated with higher mortality rates [6, 7]. Furthermore the concept of organ preservation does not always coincide with function preservation. On the other hand, other studies have shown that the evolution of primary surgery, with the use of CO_2_ laser, robotic surgery, and microvascular reconstruction, has reduced surgery-related morbidity and mortality and improved function with even better oncologic results in some cases [8–12]. The most appropriate treatment regimen is therefore still controversial.

Prognostic factors are important in helping physicians to select the best treatment modality for the individual patient and for better planning of prospective studies. T4a tumors were first defined in the 2002 TNM staging and represented a unique study group, because although the tumor has invaded critical structures it can still be resected surgically. To the best of our knowledge, this is the largest study to assess oncologic outcome and prognostic factors in T4a OPC and also the first study to examine the role of p16 expression as a marker for HPV infection solely in this patient group [13].

## 2. Methods

A retrospective study was conducted at an academic tertiary referral center (Department of Otorhinolaryngology, Head and Neck Surgery, University of Erlangen-Nuremberg Medical School, Erlangen, Germany). Patients referred to our hospital between 1980 and 2010, who had received definitive treatment for previously untreated squamous cell carcinomas of the oropharynx, were considered for selection. The study included all patients who had a cT4a Nall M0 tumor if not treated with primary operation and pT4a if primary surgical treatment was applied (stage IVa or IVb). Exclusion criteria were previous treatment for head and neck carcinomas, distant metastases at the time of diagnosis, histology other than SCC, and patients with second primary tumors at the time of diagnosis. The institutional review board approved the study.

After reviewing the pretherapeutic imaging and the surgical and the pathology reports, staging was reevaluated according to the 2010 American Joint Committee on Cancer (AJCC) and Union Internationale Contre le Cancer (UICC) classification [14]. Since T4 carcinomas had been subdivided into T4a and T4b in 2002, the files of patients with T4 tumors treated prior to this date were carefully reassessed to differentiate between T4a and T4b. In 31 cases this differentiation was not possible and these patients were not included for further evaluation. Following clinical examination, standard diagnostic investigations included ultrasonography and computed tomography. Magnetic resonance imaging (MRI) was also used in a few cases. The appropriate treatment modality was decided by our interdisciplinary tumor board. Factors influencing the decision were the operability of the tumor, general health, and personal preference of each patient.

The endpoints for the analysis were disease-specific survival (DSS), local control (LC), and regional control (RC). DSS was defined as the time from the date of diagnosis to death from the cancer or complications of treatment. Duration of LC or RC was calculated from the date of initial diagnosis to the date of most recent clinical review when local or regional recurrence was confirmed. Local recurrence was defined as invasive carcinoma at the anatomic site of the primary tumor and regional recurrence as invasive carcinoma in the lymph nodes of the neck, developing after completion of the initial treatment. Calculations of five-year overall survival (OS), disease-specific survival (DSS), local control (LC), and regional control (RC) were made with Kaplan-Meier estimates and compared using the log-rank test. A *P* value of less than 0.05 was considered significant. Multivariate analysis was performed with backward stepwise Cox regression using significant variables from the univariate analysis. All statistical analyses were performed using SPSS Version 20 (SPSS Inc., Chicago IL, USA).

Any HPV infection in tumor tissue was determined retrospectively, using p16 immunohistochemistry as a highly sensitive and specific surrogate marker for HPV-associated carcinogenesis [15]. P16 immunohistochemistry was performed using a primary antibody from Santa Cruz Biotechnology (clone JC8, dilution: 1 : 100). Tumors were considered positive for p16 when strong nuclear and cytoplasmic staining was present in >60% of cells. The p16 oncoprotein expression was successfully determined using paraffin blocks available from 93 patients treated between 2000 and 2010. Characteristics of the two groups were controlled using the chi-square test.

## 3. Results

Initially 581 Patients were selected. Forty-five cases were excluded because of second primary tumors at the time of diagnosis, 53 patients because of distant metastases, and 57 patients because of incomplete treatment. The final study population consisted of 426 patients who met the inclusion criteria. There were 374 men and 52 women, with 7.2 : 1 male to female ratio. The median age at presentation was 54 years ranging from 32 to 82 (SD 9.9). Median follow-up was 1.64 years (range: 0–26.3). For surgically treated patients, median follow-up was 1.54 years (range: 0–17.2) and for nonsurgically treated patients follow-up was 2.11 years (range: 0–26.3). 245 (57.5%) patients were smokers, 51 (11.9%) exsmokers, and 21 (4.9%) nonsmokers. Information about smoking was not available for 109 (25.6%) patients. 237 (55.6%) patients drank alcohol, 48 (11.3%) were exdrinkers, and 28 (6.6%) teetotalers. No information was available for 115 patients.

The five-year OS was 21.6% (95% CI: 17–26%), DSS 35.6% (95% CI: 30–41%); LC was seen in 81.3% (95% CI: 77–86%) and RC in 89% (95% CI: 84–94%). There was a local recurrence in 65 (15.6%) patients, a regional one in 22 (5.16%), and distant metastasis in 27 (6.3%) of the 426 patients. Mean time to the first local recurrence was 0.47 years.

**Figure 1:**
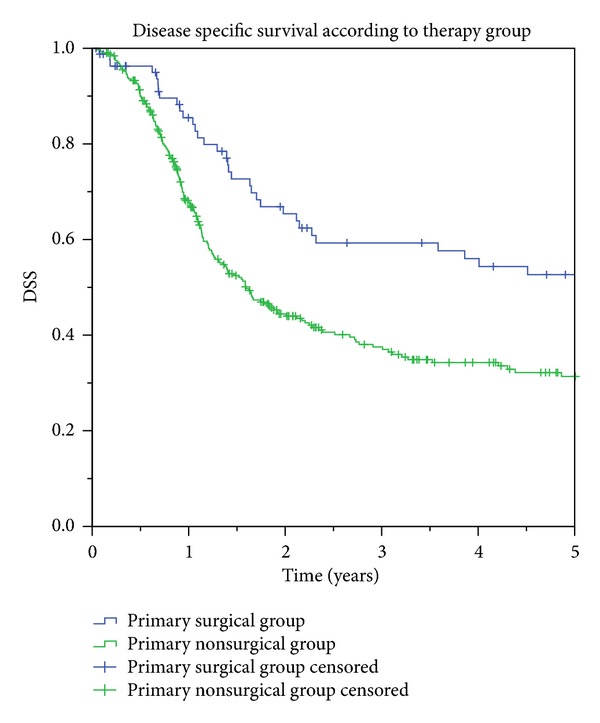
DSS (disease-specific survival) estimates according to treatment group (52.7% 95% CI 41–65% versus 31.4% 95% CI 23–39%, *P* = 0.001).

Two major groups were defined according to the type of management. The first group consisted of 316 patients who received radiotherapy with or without concomitant chemotherapy (RCT group) and salvage surgery in some cases. The second group of 83 patients received primary surgical treatment with or without adjuvant radio- (or chemo)therapy.  Table 1 shows survival estimates in relation to treatment modality. The prognosis was found to differ significantly between the two groups. Univariate analysis showed that the surgery group had a significantly better DSS (*P* < 0.001) and OS (*P* < 0.001) in comparison with the RCT group. On the other hand, LC and RC were comparable in the two groups.  Figure 1 presents the Kaplan-Meier curve of DSS in relation to the primary treatment group. A specific comparison of only those cases with combined treatment modalities showed that both DSS and OS were statistically better following surgery with adjuvant radio- or radiochemotherapy (64 patients) in comparison with combined primary chemoradiotherapy with or without salvage surgery (151 patients) (OS = 44.4% [95% CI: 32–57%] versus 18.6% [95% CI: 12–25%], *P* < 0.001) (DSS = 53.5% [95% CI: 40–67%] versus 33.8% [95% CI: 24–42], *P* < 0.001). Twenty-seven patients could not be included in either of the two groups because they had only palliative therapy (e.g., only chemotherapy or postchemotherapy salvage surgery).

Evidence of regional disease was found in 303 cases.  Table 2 shows survival according to the clinical N category. For the statistical analysis, patients were grouped in cN0 and cN+ cases. As shown in Table 4, patients who were cN0 had significantly better DSS and OS. Of the patients classified as cN0 who underwent primary surgical therapy (13), 9 had an elective neck dissection. Three out of nine patients had evidence of regional metastases on histopathology, giving an occult metastasis rate of 33.3%.

Immunohistochemical p16 oncoprotein expression was determined in 93 patients. Fifteen proved to be p16-positive, while 78 were p16-negative, giving a rate of 16%. The mean age of the former group was 56.2 and that of the latter was 62.8 years. Univariate analysis revealed a better DSS (64.6% versus 40.2%, *P* = 0.126) and OS (40.2% versus 23.4%, *P* = 0.388) for p16-positive cases but the differences were not statistically significant.  Table 3 shows the oncologic results in relation to the therapy group for p16-positive and p16-negative patients separately. Three cases could not be included in either of the treatment groups, so that 90 patients were analyzed. The small number of p16-positive patients did not allow for a statistical comparison of the two treatment groups. On the other hand, patients who were p16-negative showed a trend towards better survival following primary surgery than after primary conservative treatment. Figures for OS were 50.0% versus 19.1% (*P* = 0.102) and for DSS were 71.4% versus 34.0% (*P* = 0.113), respectively.

Of the 83 patients who underwent primary surgical treatment, clear surgical margins (R0) were achieved in 64 patients (77.1%), while resections had positive margins (R+) in the remaining 19 patients (22.9%). As Table 4 shows, patients with R0 resections had better DSS, OS, and distal metastasis rates. Nineteen of the surgical patients did not have a neck dissection, however, and they showed significantly worse DSS and OS.

Table 4 shows the results of univariate analysis of eleven potential prognostic factors. Statistical analysis of perinodal and lymphatic invasion was flawed because of the small number of cases in one group. Nevertheless, patients without perinodal invasion had better DSS and OS. Female patients showed a trend towards better DSS and OS. Lastly, age and tumor differentiation did not affect survival. The base of the tongue and the tonsillar region (i.e., tonsil, tonsillar fossa, and pillars) were the most commonly affected subsites. As seen in Table 4, univariate analysis did not reveal any significant differences in survival according to the affected anatomic subsite.

A multivariate analysis of appropriate variables was then performed. The N category proved to be a statistically significant independent predictor of reduced DSS (OR = 2.662;* P* (Wald) = 0.001; 95% CI 1.709 to 4.145) and OS (OR = 2.255;* P* (Wald) = 0.003; 95% CI 1.327 to 3.834).

## 4. Discussion

The incidence of OPSCC has been increasing continuously in recent years, a development that has been attributed mainly to HPV infection [16, 17]. Although advances in high-precision radiotherapy and new systemic agents have made nonsurgical treatment of advanced OPSCC, the standard care in many centers, the most appropriate treatment regimen is still the subject of debate [18–20]. The prognostic value of HPV infection has gained importance in clinical research, especially of OPSCC [16, 21]. The expression of p16, which is a surrogate marker for HPV, is usually measured [17]. Nevertheless, a large proportion of patients have HPVnegative OPSCC and, for this reason, their optimal treatment modality should not be neglected [22].

In our study, OPSCC with probably HPV-related pathogenesis represented 16% of cases; this is lower than in recent studies [21] which showed proportions of 40.5–72.2% [23, 24]. There are two explanations for this phenomenon: first, the relatively low proportion of HPV-related oropharyngeal carcinoma in Germany compared to other regions [25] and second, the advanced T category. Patients with HPV-related carcinoma are younger and more discerning and therefore seek medical help before the tumor becomes locally advanced (T4a) [17]. In consequence, the majority of patients with T4a carcinoma treated in Germany are HPV-negative OPSCC. Our study is consistent with previous work that demonstrated worse survival rates for HPV-negative patients [5, 21, 25]. Although our results were not statistically significant, this can be attributed to the small number of p16-positive patients. In fact the low percentage of patients with information about p16 status (93/426) is a weakness of this study.

The poor survival of HPV-negative patients with locally advanced OPSCC makes it essential that treatment modalities be improved for this large patient group, as the optimal treatment regimen has yet to be found. This is in contrast to patients with HPV-positive tumor or with early OPSCC, for whom deescalation of treatment is being discussed [26, 27]. A prospective study comparing primary surgical treatment and primary conservative treatment is unlikely to be realized, since patient and clinician preferences would make recruitment almost impossible. As long as prospective randomized data are lacking, however, nonrandomized data, such as those presented in our study, may offer a basis for treatment decision making.

Our data show a trend toward better OS and DSS for the primary surgically treated patients with HPV-negative T4a OPSCC. Of course, it could be argued that there is a selection bias in the two treatment modalities. In fact the major weakness of the study is the selection bias in a historical cohort covering a 30-year era of various types and protocols in the treatment decision making. Therefore, this study should not be considered as a direct comparison between surgery and RCT. The study does, however, present evidence that primary surgery might have a clear role in advanced HPV-negative OPSCC. Other studies have also shown a survival benefit in surgical patients [19, 28]. In the case of primary surgery, our study confirmed the prognostic impact of clear resection margins (R0), emphasizing their importance in survival [29]. The high percentage of incomplete tumor resection in our study (22.9%) can partially be explained by inadequate pathologic assessment of frozen sections and surgical approach and emphasizes the need for the careful preoperative selection of patients and the painstaking surgical technique required to optimize oncologic results. Furthermore the high percentage of nonsurgically treated patients in this study (316/426) shows that primary radiochemotherapy remains the treatment of choice in most cases with advanced OPSCC.

Another promising therapeutic alternative currently being investigated for advanced OPSCC is the use of induction chemotherapy followed by concomitant chemoradiation or surgery [30–32]. In a recent phase III trial, the additional use of panitumumab in patients receiving primary radiotherapy and cisplatin significantly improved survival in HPV-negative patients [33].

Prognostic factors are important in selecting the appropriate treatment for the patient. Furthermore, prognostic factors can help proper stratification in future randomized trials. Our study investigated eleven possible prognostic factors for the oncologic outcome in T4a oropharyngeal carcinoma. The strongest prognostic factor for T4a carcinomas in univariate and multivariate analysis proved to be the presence of lymph node metastases. Perhaps future trials could investigate the oncologic safety of deescalation of treatment in HPV-positive patients with cN0 neck. The incidence of occult metastases was 33%, which is comparable to previous studies and confirms the need for elective treatment of the neck [34–36]. Perinodal invasion was also shown to be a significant prognostic factor but the widely different group sizes reduced the power of the statistical analysis. Interestingly, recent studies could not verify the prognostic importance of extracapsular spread in HPV-positive OPSCC, and the authors concerned suggest deescalating adjuvant therapy in this patient group, even if there is evidence of ECS [27].

In the present study, 155 of the initial 581 patients were excluded because they did not meet the inclusion criteria. Fifty-seven (19.8%) of them were excluded because they were not able to receive proper treatment. This percentage is comparable with the literature and shows that many patients with advanced OPSCC are not able to receive the intended curative treatment, a problem that is often ignored in many studies [37].

## 5. Conclusion

HPV-positive cases seem to account for a low proportion of T4a OPSCC in our patient cohort (16%) and further studies should investigate if this percentage increases with time. HPV-negative patients, on the other hand, had worse survival but performed better after primary surgical treatment. The strongest independent prognostic factor for T4a carcinomas in multivariate analysis proved to be the presence of cervical lymph node metastases (pN+).

## 6. Synopsis

The strongest independent prognostic factor for T4a carcinomas proved to be the presence of lymph node metastases. The survival of p16-negative patients was worse than p16-positive patients, although they seem to respond better to primary surgery.

## Figures and Tables

**Table 1:** Oncologic results according to treatment group.

Treatment group	Number of patients	5-Y-KM-Estimate (%) (total number of events) (95% CI)				
		OS	DSS	LC	RC	DC
Primary surgical group	**83**	**41.2 (67) (30**–**52)**	**52.7 (35) (41**–**65)**	**74.2 (16) (63**–**86)**	*84.7 (8) (75–**95)**	*86.5 (6) (76–**97)**
Surgery	19	30.4 (16) (6–54)	*47.2 (8) (19–75)	*55.6 (5) (26–85)	*67.5 (2) (27–100)	No events*
Surgery + RT	38	38.9 (33) (21–52)	41.8 (20) (25–58)	*75.7 (8) (6–91)	*81.6 (5) (67–96)	*90.0 (2) (76–100)
Surgery + RCT	26	56.7 (18) (37–76)	*72.7 (7) (54–92)	*87.5 (3) (71–100)	*95.8 (1) (87–100)	56.7 (18) (37–76)
Primary conservative group	**316**	**17.3 (263) (13**–**22)**	**31.4 (184) (23**–**39)**	**83.8 (43) (79**–**89)**	**91.8 (11) (86**–**97)**	**80.4 (20) (71**–**89)**
RCT ± salvage	151	18.6 (129) (12–25)	33.8 (91) (24–42)	83.5 (21) (76–91)	*96.0 (4) (92–100)	76.8 (11) (62–91)
RT ± salvage	165	16.8 (134) (11–23)	29.6 (93) (21–38)	84.2 (22) (78–91)	*88.8 (7) (80–98)	83.0 (9) (71–95)

*Not enough events or patients for statistical analysis.

**Table 2:** Oncologic results according to cN category.

N category	Number of patients	5-Y-KM-Estimate (%) (Total number of events) (95% CI)				
		OS	DSS	LC	RC	DC
N0	115	40.3 (85) (31–50)	57.1 (43) (47–67)	83.5 (18) (76–91)	*91.5 (6) (84–99)	*85.7 (9) (77–95)
N1	35	17.1 (31) (5–30)	31.7 (22) (14–49)	*80.0 (7) (67–93)	*75.9 (3) (50–1)	No events*
N2	222	13.6 (191) (9–19)	27.7 (132) (20–35)	79.6 (31) (71–88)	*91.9 (8) (86–97)	79.3 (15) (68–91)
N3	46	15.8 (40) (5–27)	20.5 (32) (8–33)	*80.8 (7) (67–94)	*80.7 (5) (63–99)	*76.0 (3) (51–100)
N*x*	8	*25.0 (7) (0–55)	*33.3 (5) (0–71)	*72.9 (2) (41–1)	No events*	No events*

**Table 3:** Oncologic results according to HPV infection and treatment group.

p16	Treatment group (number of patients)	5-Y-KM-Estimate (%) (Total number of events) (95% CI)				
		OS	DSS	LC	RC	DC
*p16-positive	Primary surgical group (5)	*75.0 (2) (32–100)	No events	No events	No events	*75.0 (1) (32–100)
	RCT group (10)	*26.7 (7) (0–56)	*50.0 (5) (19–81)	*90.0 (1) (71–100)	No events	*88.9 (1) (68–100)

p16-negative	Primary surgical group (10)	50.0 (6) (15–85)	*71.4 (2) (38–100)	*62.5 (2) (21–100)	No events	*85.7 (1) (60–100)
	RCT group (65)	19.1 (43) (6–33) *P* = 0.102	34.0 (30) (15–53) *P* = 0.113	*86.2 (8) (77–95) *P* = 0.79	*97.0 (1) (90–100) *P* = 0.67	*67.5 (5) (35–100) *P* = 0.81

All (90)		26.9 (58) (15–38)	44.5 (37) (30–59)	79.6 (11) (65–94)	*97.9 (1) (94–100)	*76.8 (8) (58–95)

*Not enough events or patients for statistical analysis.

**Table 4:** Univariate analysis of potential prognostic factors for DSS, OS, LC, RC and distant metastases.

Variable (number of patients)	KM estimates, total number of events, and 95% CI concerning defined events 5-Y-KM-Estimate % (total number of events) (95% CI)				
	Local control (LC)	Neck control (NC)	Distant metastases	Disease-specific survival (DSS)	Overall survival (OS)
Age (years)					
≤54 (223)	77.0 (39) (70–84)	90.1 (10) (83–97)	81.6 (18) (73–90)	31.3 (134) (24–38)	20.8 (190) (15–26)
>54 (203)	85.3 (26) (80–91)	87.6 (12) (80–95)	86.1 (9) (77–95)	41.0 (100) (33–49)	22.5 (164) (16–29)
	*P* = 0.199	*P* = 0.495	*P* = 0.127	*P* = 0.165	*P* = 0.864
Gender					
Male (374)	80.4 (59) (75–85)	87.6 (21) (82–93)	82.7 (23) (75–90)	34.0 (212) (28–40)	19.9 (313) (16–24)
Female (52)	*87.0 (6) (77–97)	*89.1 (1) (84–94)	*86.6 (4) (74–99)	47.5 (100) (42–73)	32.8 (41) (20–46)
	^ +^ *P* = 0.392	^ +^ *P* = 0.207	^ +^ *P* = 0.942	^ +^ *P* = 0.074	^ +^ *P* = 0.188
Tumor subsite					
Tonsillar region (173)	82.8 (26) (76–89)	83.5 (10) (73–94)	80.4 (14) (70–91)	32.0 (99) (24–40)	19.3 (147) (13–25)
Soft palate (25)	*72.2 (5) (49–95)	*95.0 (1) (85–100)	*635 (3) (25–100)	44.6 (13) (24–65)	21.0 (21) (04–38)
Base of the tongue (189)	81.4 (28) (74–89)	91.1 (10) (86–97)	*88.3 (8) (80–97)	36.2 (103) (28–44)	23.9 (154) (17–30)
Posterior wall (39)	*81.2 (6) (67–95)	*95.5 (1) (87–100)	*86.7 (2) (69–100)	43.3 (19) (26–61)	21.5 (32) (08–35)
R status					
R0 (64)	80.0 (12) (70–90)	82.2 (8) (71–94)	91.4 (3) (82–100)	60.9 (23) (48–74)	47.2 (52) (35–60)
R+ (19)	39.4 (4) (15–65)	— (0) (—; —)	65.5 (3) (33–98)	23.5 (12) (01–46)	20.1 (15) (01–40)
	^ +^ *P* = 0.466		^ +^ *P* = 0.027	^ +^ *P* = 0.001	^ +^ *P* = 0.003
N category					
N0 (115)	83.5 (18) (76–91)	*91.5 (6) (84–99)	*85.7 (9) (77–95)	57.1 (43) (47–67)	40.3 (85) (31–50)
N+ (303)	80.3 (45) (74–87)	87.2 (16) (80–94)	81.8 (18) (73–91)	26.9 (186) (21–33)	14.5 (262) (10–19)
	^ +^ *P* = 0.715	^ +^ *P* = 0.269	^ +^ *P* = 0.352	*P* < 0.001	*P* < 0.001
ND (surgical therapy)					
No (19)	*65.9 (5) (37–93)	*78.6 (2) (51–100)	*— (0) (—; —)	22.6 (13) (01–44)	15.8 (17) (00–32)
Yes (64)	79.4 (11) (68–91)	*86.0 (6) (75–97)	*84.5 (6) (73–96)	61.1 (22) (48–74)	49.3 (50) (37–62)
	^ +^ *P* = 0.134	^ +^ *P* = 0.382	^ +^ *P* = 0.327	^ +^ *P* = 0.001	^ +^ *P* = 0.022
Differentiation					
Well/moderate (264)	82.4 (39) (77–88)	88.8 (14) (83–95)	82.2 (16) (73–91)	34.1 (147) (27–41)	20.6 (221) (16–26)
Poor (125)	80.7 (19) (71–90)	*91.5 (6) (85–98)	83.2 (10) (73–93)	38.8 (59) (29–49)	22.5 (102) (15–30)
	*P* = 0.966	*P* = 0.697	*P* = 0.649	*P* = 0.398	*P* = 0.368
Perinodal invasion					
No (153)	78.3 (30) (71–86)	88.4 (11) (82–95)	84.7 (12) (76–93)	51.7 (62) (42–61)	34.6 (117) (27–43)
Yes (21)	*65.9 (3) (62–70)	*79.5 (2) (54–100)	*86.3 (2) (68–100)	19.9 (11) (01–43)	13.2; (16) (0–29)
	^ +^ *P* = 0.814	^ +^ *P* = 0.275	^ +^ *P* = 0.262	^ +^ *P* = 0.011	^ +^ *P* = 0.052
Lymphatic invasion					
No (164)	79.1 (31) (72–86)	88.2 (12) (82–95)	84.8 (13) (77–93)	50.8 (68) (42–60)	33.2 (128) (26–41)
Yes (17)	*64.3 (3) (25–100)	*87.5; (1) (65–100)	*91.7; (1) (76–100)	*30.0 (7) (00–63)	17.9 (11) (04–39)
	^ +^ *P* = 0.836	^ +^ *P* = 0.682	^ +^ *P* = 0.733	^ +^ *P* = 0.126	^ +^ *P* = 0.292
Treatment					
Primary surgery (83)	74.2 (16) (63–86)	*84.7 (8) (75–95)	*86.5 (6) (76–97)	52.7 (35) (41–65)	41.2 (67) (30–52)
Primary nonsurgical treatment (316)	83.8 (43) (79–89)	91.8 (11) (86–97)	80.4 (20) (71–89)	31.4 (184) (23–39)	17.3; (263) (13–22)
	^ +^ *P* = 0.420	^ +^ *P* = 0.102	^ +^ *P* = 0.529	^ +^ *P* = 0.001	^ +^ *P* = 0.001
HPV					
No (78)	76.6 (10) (59–94)	*97.4 (1) (93–100)	*75.3 (6) (53–98)	40.2 (33) (24–56)	23.4 (52) (11–35)
Yes (15)	*93.3 (1) (81–100)	*— (0) (—; —)	*82.1 (2) (59–100)	*64.6 (5) (40–90)	*40.2 (9) (13–68)
	^ +^ *P* = 0.440	^ +^ *P* = 0.631	^ +^ *P* = 0.711	^ +^ *P* = 0.126	*P* = 0.388

*Not enough events or patients for statistical interpretation.

^+^Too widely different group sizes or crossings for interpretation without a comment.

## Acronyms

OPSCC: Oropharyngeal squamous cell carcinoma
RT: Radiotherapy
RCT: Radiochemotherapy
SCC: Squamous cell carcinoma
AJCC: American Joint Committee on Cancer
UICC: Union Internationale Centre Contre Cancer
DSS: Disease-specific survival
LC: Local control
RC: Regional control
OS: Overall survival
HPV: Human papillomavirus.

## References

[1] Parsons JT, Mendenhall WM, Stringer SP (2002). Squamous cell carcinoma of the oropharynx: Surgery, radiation therapy, or both. *Cancer*.

[2] Allal AS, Nicoucar K, Mach N, Dulguerov P (2003). Quality of life in patients with oropharynx carcinomas: assessment after accelerated radiotherapy with or without chemotherapy versus radical surgery and postoperative radiotherapy. *Head and Neck*.

[3] Mowry SE, Ho A, Lotempio MM, Sadeghi A, Blackwell KE, Wang MB (2006). Quality of life in advanced oropharyngeal carcinoma after chemoradiation versus surgery and radiation. *Laryngoscope*.

[4] Ihloff AS, Petersen C, Hoffmann M, Knecht R, Tribius S (2010). Human papilloma virus in locally advanced stage III/IV squamous cell cancer of the oropharynx and impact on choice of therapy. *Oral Oncology*.

[5] Ang KK, Harris J, Wheeler R (2010). Human papillomavirus and survival of patients with oropharyngeal cancer. *The New England Journal of Medicine*.

[6] Pederson AW, Haraf DJ, Witt M-E (2010). Chemoradiotherapy for locoregionally advanced squamous cell carcinoma of the base of tongue. *Head and Neck*.

[7] Eisbruch A, Harris J, Garden AS (2010). Multi-institutional trial of accelerated hypofractionated intensity-modulated radiation therapy for early-stage oropharyngeal cancer (RTOG 00–22). *International Journal of Radiation Oncology Biology Physics*.

[8] Weinstein GS, O'Malley BW, Magnuson JS (2012). Transoral robotic surgery: a multicenter study to assess feasibility, safety, and surgical margins. *Laryngoscope*.

[9] Bozec A, Poissonnet G, Chamorey E (2009). Quality of life after oral and oropharyngeal reconstruction with a radial forearm free flap: prospective study. *Journal of Otolaryngology*.

[10] Psychogios G, Mantsopoulos K, Kuenzel J (2012). Primary surgical treatment of T2 oropharyngeal carcinoma. *Journal of Surgical Oncology*.

[11] Kunzel J, Iro H, Psychogios G, Zenk J, Koch M (2013). Closure of defects after resection of tumors of the oral cavity and the pharynx: medium- to long-term oncologic and functional results with the myocutaneous platysma flap. *European Archives of Oto-Rhino-Laryngology*.

[12] Ryzek DF, Künzel J, Grundtner P, Zenk J, Iro H, Psychogios G (2014). Early stage oropharyngeal carcinomas: comparing quality of life for different treatment modalities. *BioMed Research International*.

[13] Haughey BH, Sinha P (2012). Prognostic factors and survival unique to surgically treated p16+ oropharyngeal cancer. *Laryngoscope*.

[14] American Joint Committee on Cancer (AJCC) (2010). *Cancer Staging Manual*.

[15] Ukpo OC, Flanagan JJ, Ma XJ, Luo Y, Thorstad WL, Lewis JS (2011). High-risk human papillomavirus E6/E7 mRNA detection by a novel in situ hybridization assay strongly correlates with p16 expression and patient outcomes in oropharyngeal squamous cell carcinoma. *The American Journal of Surgical Pathology*.

[16] Wittekindt C, Wagner S, Mayer CS, Klußmann JP (2012). Basics of tumor development and importance of human papilloma virus (HPV) for head and neck cancer. *Laryngo-Rhino-Otologie*.

[17] Psychogios G, Alexiou C, Agaimy A (2014). Epidemiology and survival of HPV related tonsillar carcinoma. *Cancer Medicine*.

[18] Loewenthal M, Vitez E, Laban S (2012). New aspects of current therapeutic strategies in oropharyngeal carcinoma: highlights of the 2012 ASCO meeting. *HNO*.

[19] Karatzanis AD, Psychogios G, Mantsopoulos K (2012). Management of advanced carcinoma of the base of tongue. *Journal of Surgical Oncology*.

[20] Garden AS, Kies MS, Morrison WH (2013). Outcomes and patterns of care of patients with locally advanced oropharyngeal carcinoma treated in the early 21st century. *Radiation Oncology*.

[21] Mehanna H, Olaleye O, Licitra L (2012). Oropharyngeal cancer—is it time to change management according to human papilloma virus status?. *Current Opinion in Otolaryngology and Head and Neck Surgery*.

[22] Marklund L, Nasman A, Ramqvist T, Dalianis T, Munck-Wikland E, Hammarstedt L (2012). Prevalence of human papillomavirus and survival in oropharyngeal cancer other than tonsil or base of tongue cancer. *Cancer Medicine*.

[23] Mehanna H, Beech T, Nicholson T (2013). Prevalence of human papillomavirus in oropharyngeal and nonoropharyngeal head and neck cancer-systematic review and meta-analysis of trends by time and region. *Head and Neck*.

[24] Panwar A, Batra R, Lydiatt WM, Ganti AK (2014). Human papilloma virus positive oropharyngeal squamous cell carcinoma: a growing epidemic. *Cancer Treatment Reviews*.

[25] Semrau R, Duerbaum H, Temming S (2013). Prognostic impact of human papillomavirus status, survivin, and epidermal growth factor receptor expression on survival in patients treated with radiochemotherapy for very advanced nonresectable oropharyngeal cancer. *Head and Neck*.

[26] Psychogios G, Mantsopoulos K, Agaimy A (2013). Prognostic factors in limited (T1-2, N0-1) oropharyngeal carcinoma treated with surgery +/- adjuvant therapy. *Head and Neck*.

[27] Sinha P, Lewis JS, Piccirillo JF, Kallogjeri D, Haughey BH (2012). Extracapsular spread and adjuvant therapy in human papillomavirus-related, p16-positive oropharyngeal carcinoma. *Cancer*.

[28] Rades D, Seibold ND, Gebhard MP, Noack F, Schild SE, Thorns C (2011). Prognostic factors (including HPV status) for irradiation of locally advanced squamous cell carcinoma of the head and neck (SCCHN). *Strahlentherapie und Onkologie*.

[29] Mantsopoulos K, Psychogios G, Waldfahrer F, Zenk J, Iro H (2012). Surgical treatment of locally limited tonsillar cancer. *Surgical Oncology*.

[30] Masterson L, Tanweer F (2013). The role of sequential chemoradiation for local advanced oropharyngeal carcinoma. *International Journal of Clinical Oncology*.

[31] Oertel K, Spiegel K, Schmalenberg H (2012). Phase I trial of split-dose induction docetaxel, cisplatin, and 5-fluorouracil (TPF) chemotherapy followed by curative surgery combined with postoperative radiotherapy in patients with locally advanced oral and oropharyngeal squamous cell cancer (TISOC-1). *BMC Cancer*.

[32] Worden FP, Kumar B, Lee JS (2008). Chemoselection as a strategy for organ preservation in advanced oropharynx cancer: response and survival positively associated with HPV16 copy number. *Journal of Clinical Oncology*.

[33] Licitra L, Bergamini C, Mirabile A, Granata R (2011). Targeted therapy in head and neck cancer. *Current Opinion in Otolaryngology and Head and Neck Surgery*.

[34] Psychogios G, Mantsopoulos K, Bohr C, Koch M, Zenk J, Iro H (2013). Incidence of occult cervical metastasis in head and neck carcinomas: development over time. *Journal of Surgical Oncology*.

[35] Fasunla AJ, Greene BH, Timmesfeld N, Wiegand S, Werner JA, Sesterhenn AM (2011). A meta-analysis of the randomized controlled trials on elective neck dissection versus therapeutic neck dissection in oral cavity cancers with clinically node-negative neck. *Oral Oncology*.

[36] Kau RJ, Alexiou C, Stimmer H, Arnold W (2000). Diagnostic procedures for detection of lymph node metastases in cancer of the larynx. *ORL*.

[37] Lybak S, Liavaag PG, Monge OR, Olofsson J (2011). Surgery and postoperative radiotherapy a valid treatment for advanced oropharyngeal carcinoma. *European Archives of Oto-Rhino-Laryngology*.

